# Prolonged low-dose tPA ameliorates coagulopathy and organ injury in an LPS-induced rat DIC model

**DOI:** 10.1007/s12185-026-04187-1

**Published:** 2026-03-09

**Authors:** Rina Takenaka, Momoka Tomiyama, Hiroaki Watanabe, Shinya Yamada, Eriko Morishita, Yukio Suga, Hidesaku Asakura

**Affiliations:** 1 Division of Pharmacy, Graduate School of Medical Sciences, Kanazawa University, Kakumamachi, Kanazawa, Ishikawa 920-1192 Japan; 2 Department of Clinical Pharmacy and Healthcare Sciences, Faculty of Pharmacy, Institute of Medical, Pharmaceutical & Health Sciences, Kanazawa University, Kakumamachi, Kanazawa, Ishikawa 920-1192 Japan; 3 Department of Hematology, Kanazawa University Hospital, 13-1 Takaramachi, Kanazawa, Ishikawa 920-8641 Japan

**Keywords:** Disseminated intravascular coagulation, Lipopolysaccharide, Tissue plasminogen activator

## Abstract

Currently, no established treatments for disseminated intravascular coagulation (DIC) specifically target fibrinolysis. We previously demonstrated that prophylactic administration of tissue plasminogen activator (tPA) to a lipopolysaccharide (LPS)-induced rat DIC model improved DIC pathophysiology. However, the optimal duration of tPA administration and its effectiveness when administered therapeutically remain unclear. In the present study, we investigated whether tPA remains effective when administered at the same dosage over different durations, and whether therapeutic administration is also effective. We found that both prophylactic and therapeutic administration of tPA increased D-dimer levels, reduced serum creatinine and the renal glomerular fibrin deposition rate, suppressed the formation of thrombin–antithrombin complex and interleukin-6, and attenuated decreases in platelet count. Furthermore, with both prophylactic and therapeutic administration of tPA, most markers of DIC pathophysiology demonstrated greater improvements with longer administration of tPA, from 15 min to 8 h. No bleeding tendency was observed based on urinary hemoglobin levels. These results suggest that a lower tPA dose rate and longer duration of administration may enhance efficacy and safety in the LPS-induced rat DIC model. A reduced dosage and extended duration of tPA administration could represent a new treatment option for clinical DIC and warrants further investigation.

## Introduction

Disseminated intravascular coagulation (DIC) is a serious condition in which significant activation of coagulation occurs in the presence of underlying disease and multiple microthrombi develop, mainly in microvessels throughout the body. In advanced cases, multiple organ failure occurs due to the microcirculatory disturbance caused by multiple microthrombi [1, 2]. On the other hand, bleeding symptoms may arise due to consumptive coagulopathy and hemostatic thrombolysis secondary to fibrinolytic activation. The degree of fibrinolytic activation in DIC varies considerably, depending on the underlying pathology [3, 4]. DIC progresses sequentially from pre-DIC, where subtle laboratory abnormalities are present, but overt DIC has not yet developed, through early-phase DIC, where abnormal coagulation biomarkers may be detected but major clinical symptoms are not yet apparent. However, in sepsis-associated DIC, levels of plasminogen activator inhibitor-1 (PAI-1), an inhibitor of fibrinolysis, become markedly increased from pre-DIC stage, and fibrinolysis is suppressed, impeding the removal of microthrombi and increasing the risk of microcirculatory disturbance. The result is that ischemic organ damage, one of the two major symptoms of DIC alongside bleeding, becomes more likely. This type of DIC is called “suppressed-fibrinolytic-type DIC” [3, 4]. On the other hand, in DIC associated with aortic aneurysm or acute promyelocytic leukemia, levels of PAI-1 are almost normal, and marked activation of fibrinolysis is observed. With this form of DIC, the other major symptom of bleeding tends to occur, while organ symptoms are rare. This type of DIC is called “enhanced-fibrinolytic-type DIC” [3, 4].

In rat models of DIC, lipopolysaccharide (LPS) and tissue factor (TF) have been frequently used to induce DIC. Our studies have revealed significant differences in the pathophysiology of DIC between the two models [3, 5, 6]. In the LPS-induced DIC model, fibrinolysis is suppressed (PAI-1 levels are elevated), and organ damage is likely [7, 8]. On the other hand, the TF-induced DIC model shows marked activation of fibrinolysis and bleeding symptoms are more likely, but organ damage is rare [9, 10]. In other words, the LPS-induced DIC model resembles the clinical situation of fibrinolysis-suppressed DIC, whereas the TF-induced DIC model resembles fibrinolysis-enhanced DIC [3].

Achieving control of DIC requires prevention of the pronounced activation of coagulation that forms its primary feature. The primary approach to DIC treatment has thus been the inhibition of thrombus formation using anticoagulants such as heparins and recombinant thrombomodulin [11, 12]. However, the development of therapies for DIC focusing on fibrinolysis as a thrombolytic mechanism has been limited.

We recently hypothesized that fibrinolytic therapy in a fibrinolysis-suppressed LPS-induced rat model of DIC could ameliorate DIC by lysing multiple microthrombi [13]. Based on this hypothesis, a previous study investigated whether administration of a tissue plasminogen activator (tPA) to this model would improve the pathophysiology of DIC. Prophylactic administration of tPA resulted in improvements to the pathophysiology of DIC, such as elevated levels of D-dimer as a fibrin degradation product, suppression of serum creatinine elevation, inhibition of glomerular fibrin deposition rate (%GFD), and mitigation of thrombocytopenia.

When tPA is administered for common thromboses such as cerebral or myocardial infarction, rapid administration is commonly employed [14]. This is because early diagnosis and prompt dissolution of the thrombus are considered crucial for improving the prognosis of patients with acute thrombosis. However, bleeding complications associated with the use of tPA remain a major challenge in clinical practice. Further, the risk of bleeding with tPA has been reported to be dose-dependent, and if tPA is rapidly administered intravenously to dissolve a thrombus quickly, as in the case of general thrombosis, the bleeding side effects would be expected to be more pronounced in DIC [15]. In other words, in the case of DIC, a shift is necessary away from the concept of "rapid" thrombus dissolution toward the anticipation of "slow" thrombus dissolution through endogenous fibrinolytic mechanisms by neutralizing PAI-1.

The present investigation extended our study of "prophylactic" tPA administration to the LPS-induced DIC model [13] described above to examine in detail whether a longer duration of tPA administration at the same dose would be more effective, and whether "therapeutic" tPA administration after completion of DIC pathophysiology would be more effective.

## Materials and methods

### Animals

Animals were maintained according to the Standards of Animal Care and Experimentation published by our institution. This study was conducted with the approval of the Kanazawa University Animal Experiment Committee (approval no. AP-204116 and AP-224307). Male Wistar rats weighed 160–210 g and were used at 7–8 weeks old (SLC Japan, Shizuoka, Japan).

### General experimental procedure

Rats were anesthetized with isoflurane (3%, 0.3 L/min, inhaled). Blood was withdrawn from the abdominal aorta into plastic syringes. All samples were diluted (1:9 v/v) with 4% sodium citrate. Rats were sacrificed by exsanguination due to blood sampling from the abdominal aorta under deep anesthesia with isoflurane (3%, 0.3 L/min, inhaled). LPS (Escherichia coli 055: B5 lipopolysaccharide; Sigma Aldrich, St. Louis, MO, USA) and Alteplase (Activacin® injection; Kyowa Kirin Co., Tokyo, Japan), used as tPA, were dissolved in physiological saline immediately before use.

**Fig. 1:**
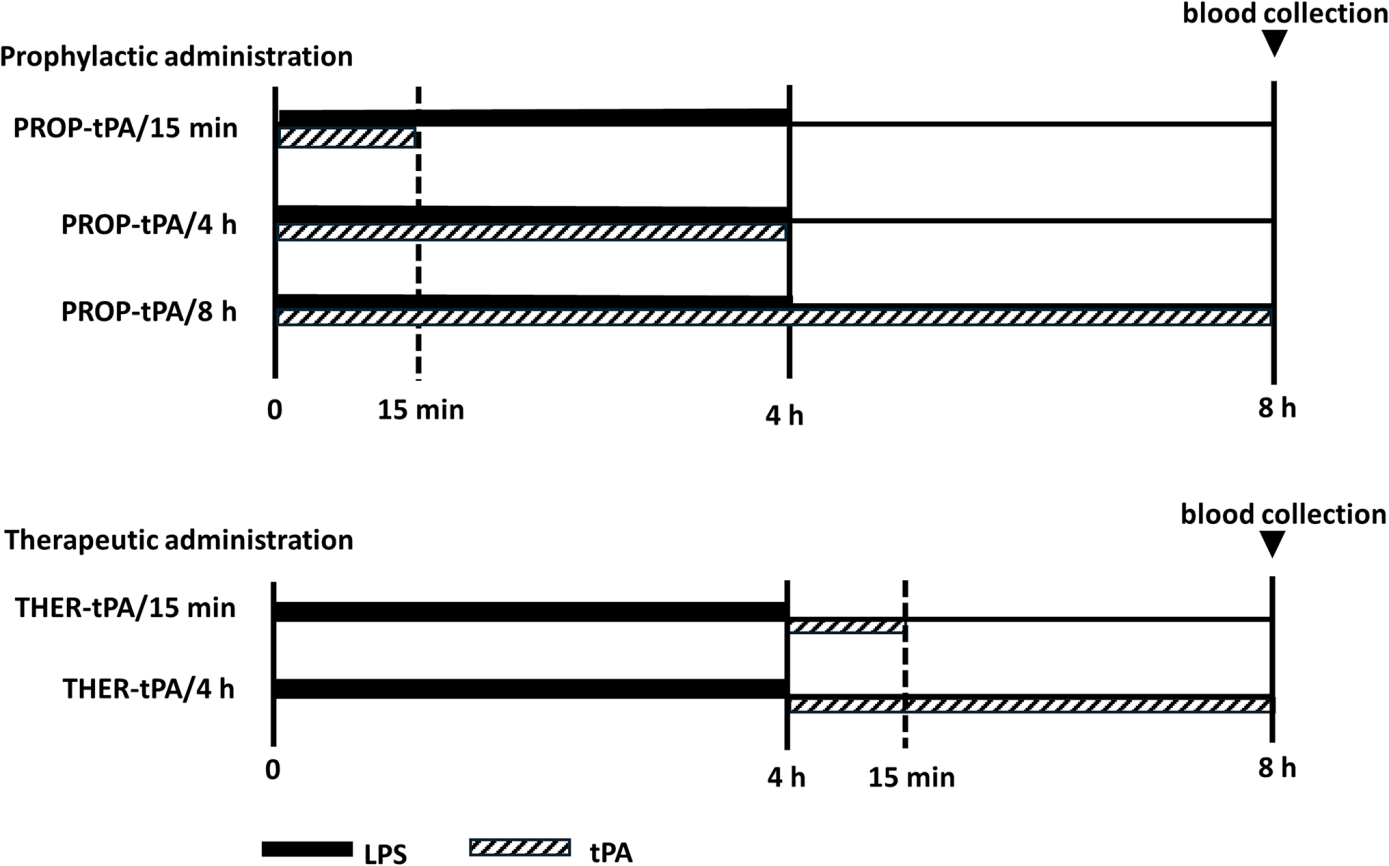
Overview of the LPS and tPA Administration Protocol

In this study, we administered tPA either prophylactically or therapeutically. The prophylactic administration groups received tPA at the same time as the initiation of LPS administration. In contrast, the therapeutic administration groups received tPA immediately after the completion of LPS administration. In the prophylactic groups, three administration times were set: 15 min, 4 h, and 8 h. In the therapeutic groups, two administration times were evaluated: 15 min and 4 h. The endpoint of this study was set at 8 h after the initiation of LPS administration to maintain the same LPS exposure duration across all groups. The prophylactic and therapeutic tPA groups were compared with the LPS group (without tPA treatment) to assess the efficacy of tPA. In addition, a group receiving physiological saline was included as a control to confirm the development of DIC induced by LPS administration. The detailed protocol is shown in Fig. 1 and described below.

Experimental DIC was induced by sustained infusion into the tail vein of 40 mg/kg LPS diluted in saline over a period of 4 h. Control group was administered a sustained 10 mL infusion of physiological saline via the tail vein over a period of 4 h. For prophylactic administration of tPA, 3 mg/kg of tPA and LPS were mixed and infused continuously through the tail vein. A 15-min infusion group received a tPA/LPS mixture at a rate of 4 mL/h for 15 min, switched to LPS-only infusion from 15 min to 4 h, and blood was collected 8 h after the start of tPA/LPS mixture administration (PROP-tPA/15 min). In the 4-h group, tPA/LPS mixture was infused at a rate of 2.5 mL/h for 4 h, and blood was drawn 8 h after starting tPA/LPS mixture administration (PROP-tPA/4 h). In the 8-h group, tPA/LPS mixture was infused at a rate of 2.5 mL/h for 4 h, switched to tPA-only infusion at a rate of 0.5 mL/h for 4 h, and blood samples were taken 8 h after starting administration of the tPA and LPS mixture (PROP-tPA/8 h). For therapeutic administration of tPA, an LPS model was created, and tPA at 3 mg/kg was infused continuously through the tail vein over either 15 min or 4 h. In the 15-min and 4-h groups, blood samples were drawn 8 h after starting LPS administration. The 15-min infusion group received LPS at rate of 2.5 mL/h for 0–4 h and tPA at a rate of 4 mL/h for 15 min (THER-tPA/15 min). The 4-h infusion group received LPS at rate of 2.5 mL/h for 0–4 h and tPA at rate of 0.5 mL/h for 4–8 h (THER-tPA/4 h). Each group consisted of 7–8 animals at each time point.

### Parameters

Platelets and urinary hemoglobin (Hb) [6]were counted using an automated device for animals (Celltac α, MEK-6558; Nihon Kohden Co., Tokyo, Japan) within 1 h of sampling. Citrated plasma samples obtained by whole blood centrifugation were stored at -80 °C until assayed. Fibrinogen concentrations were determined by clotting assays. D-dimer levels were quantified using a latex agglutination test (ELPIA ACE DD dimer; LSI Medience, Tokyo, Japan). Plasma levels of thrombin–antithrombin complex (TAT) were quantified using a commercial enzyme-linked immunosorbent assay (ELISA) kit (Enzygnost TAT micro; Behringwerke, Marburg, Germany). Plasma levels of active form of plasminogen activator inhibitor-1 (PAI-1) antigen level were quantified using rat ELISA kits (Innovative Research, USA). Interleukin-6 (IL-6) were measured using rat ELISA kits (R&D Systems, MN). To assess organ damage in rats, we measured plasma levels of creatinine (Cr) and alanine aminotransferase (ALT) using enzymatic (PureautoS CRE-L; Sekisui Medical Co., Tokyo, Japan) and spectrophotometric (Ltypewako ALT J2: FUJIFILM Wako Pure Chemical Co., Osaka, Japan) determinations, respectively.

### Histological examinations

Renal tissue specimens were collected from animals sacrificed under deep anesthesia immediately after blood sampling at 8 h after starting administration of saline or LPS, then fixed in formalin. The percentage (%) of glomerular fibrin deposition (GFD) was assessed by microscopy. After staining specimens with phosphotungstic acid hematoxylin, each sample was histologically examined by a board-certified specialist in thrombosis and hemostasis and graduate student who were blinded to the sample group. One hundred glomeruli were examined in each sample, and numbers of thrombi containing fibrin are expressed as percentages.

### Statistical analysis

Data are presented as median (range). Overall differences among groups were first assessed using the Kruskal–Wallis test. When significant differences were detected, pairwise comparisons between the LPS group and each treatment group were performed using the Mann–Whitney U test, with P-values adjusted using Holm’s method. P < 0.05 was considered statistically significant.

## Results

### Prophylactic tPA administration in the LPS model

**Fig. 2:**
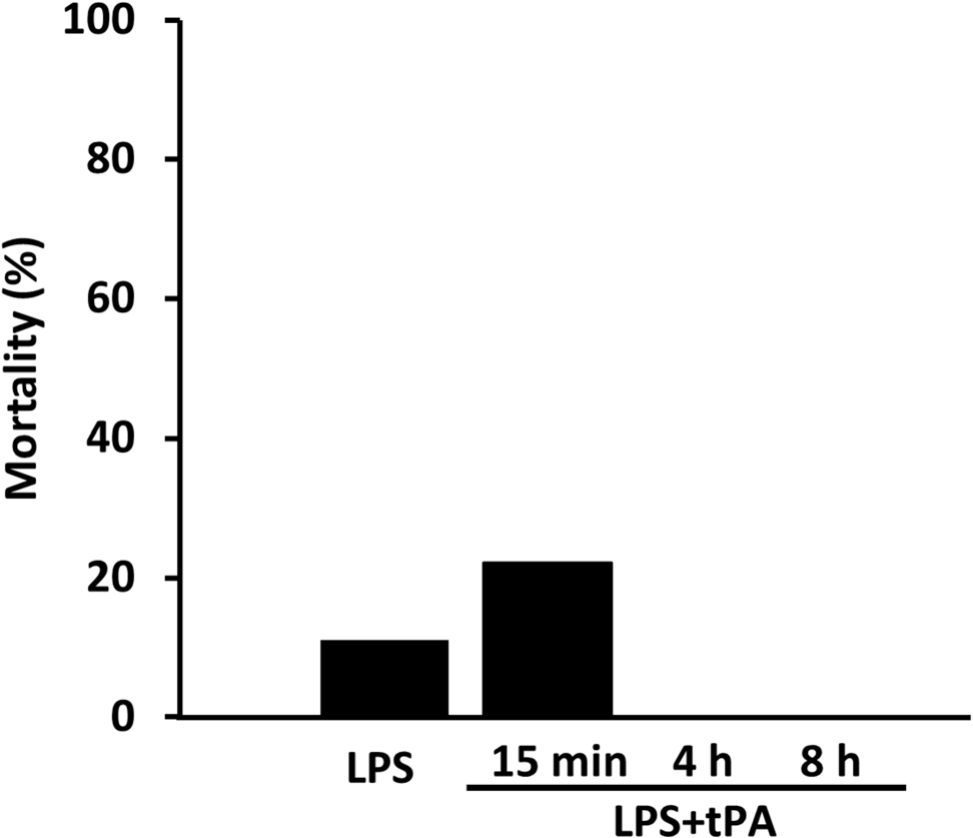
Changes in mortality at 8 h in LPS treated with prophylactic tPA. LPS was infused for a 4 h period (from 0 to 4 h). No significant differences were observed between the tPA-treated group and the LPS group. The 15-min infusion group received tPA/LPS mixture at a rate of 4 mL/h for 15 min, then switched to LPS-only infusion from 15 min to 4 h. The 4-h group received tPA/LPS mixture infused at a rate of 2.5 mL/h for 4 h. The 8-h group received tPA/LPS mixture infused at a rate of 2.5 mL/h for 4 h, then switched to tPA-only infusion at a rate of 0.5 mL/h for 4 h.

The results of prophylactic administration of tPA are shown in Table 1. Reductions in platelet count were significantly attenuated in the LPS + tPA 3 mg/kg/4 h (PROP-tPA/4 h) and LPS + tPA 3 mg/kg/8 h (PROP-tPA/8 h) groups compared to LPS group. However, the LPS + tPA 3 mg/kg/15 min (PROP-tPA/15 min) group showed no significant difference compared with the LPS group. Fibrinogen levels were significantly higher in the PROP-tPA/8 h group compared with the LPS group, but there were no significant differences in the PROP-tPA/15 min and PROP-tPA/4 h groups compared to the LPS group. PAI-1 activity levels were significantly increased in PROP-tPA/4 h group compared to LPS group, but significantly decreased in the PROP-tPA/8 h group compared to the LPS group. No significant difference in PROP-tPA/15 min was seen compared to the LPS group, despite use of the same tPA dose. D-dimer levels were significantly higher in PROP-tPA/15 min, PROP-tPA/4 h, and PROP-tPA/8 h groups compared to the LPS group. D-dimer levels progressively increased with increasing duration of tPA administration, although the tPA dose was the same. TAT levels were significantly lower in PROP-tPA/8 h compared to the LPS group, but showed no significant differences in the PROP-tPA/15 min or PROP-tPA/4 h groups compared to the LPS group. Cr levels were significantly lower in the PROP-tPA/8 h group compared with LPS group, whereas no significant difference was evident in the PROP-tPA/15 min or PROP-tPA/4 h groups compared with the LPS group. Levels of ALT did not differ significantly among PROP-tPA/15 min, PROP-tPA/4 h, and PROP-tPA/8 h groups compared with the LPS group. The %GFD was significantly lower in the PROP-tPA/8 h group compared to LPS group, whereas the PROP-tPA/15 min and PROP-tPA/4 h groups showed no significant differences compared with the LPS group. Although the difference did not reach statistical significance after multiple comparison adjustment, the PROP-tPA/8 h group showed a trend toward lower IL-6 levels compared with the LPS group (adjusted P = 0.084). Urinary Hb levels did not differ significantly among the PROP-tPA/15 min, PROP-tPA/4 h, and PROP-tPA/8 h groups compared to LPS group. Regarding mortality, an increasing trend was evident in the PROP-tPA/15 min group compared to LPS group, while a decreasing trend was seen in the PROP-tPA/4 h and PROP-tPA/8 h groups, although no significant differences were evident in either group compared to LPS group (Fig. 2).

**Table 1:** Changes in coagulation parameters, organ dysfunction and inflammatory cytokines in LPS treated with prophylactic tPA groups

Hemostatic variables	Control	LPS	LPS + tPA (3 mg/kg/15 min)	LPS + tPA (3 mg/kg/4 h)	LPS + tPA (3 mg/kg/8 h)
Platelets, × 10^3^/µL	511 (409–561)	112 (40–240)^a^	118 (78–270)	226 (122–288)^b^	267 (104–372)^b^
Fibrinogen, mg/dL	204 (176–234)	71 (50–85)^a^	55 (50–85)	57 (50–83)	94 (58–116)^b^
PAI-1, µg/L	9.1 (1.0–44.5)	859.9 (503.4–1769.1)^a^	1100.5 (381.5–2551.5)	1893.2 (1423.1–2329.8)^b^	0.3 (0.2–5.9)^b^
D-dimer, mg/L	0.20 (0.20–0.20)	0.81 (0.57–1.27)^a^	1.69 (0.38–4.56)^b^	4.42 (2.33–10.01)^b^	7.63 (3.15–11.26)^b^
TAT, µg/L	18 (6–50)	304 (262–402)^a^	289 (174–421)	331 (272–526)	180 (100–306)^b^
Cr, mg/dL	0.22 (0.18–0.26)	0.44 (0.36–0.56)^a^	0.54 (0.35–0.62)	0.41 (0.33–0.49)	0.34 (0.25–0.51)^b^
ALT, U/L	63 (25–113)	146 (75–223)^a^	221 (84–1353)	130 (113–147)	121 (61–201)
%GFD, %	0 (0–5)	53 (15–80)^a^	25 (10–55)	35 (10–40)	3 (0–10)^b^
IL-6, ng/L	82 (30–409)	61,220 (29,624–160,703)^a^	68,450 (58,569–86,024)	64,885 (45,332–68,796)	33,310 (17,192–69,649)
Urinary Hb, g/dL	0.0 (0.0–0.1)	0.0 (0.0–0.1)	0.1 (0.0–0.3)	0.0 (0.0–0.0)	0.0 (0.0–0.1)

Data are presented as median (min–max). Each group consisted of 7–8 animals

PAI-1, plasminogen activator inhibitor; TAT, thrombin–antithrombin complex; Cr, creatinine; ALT, alanine aminotransferase; IL-6, interleukin-6

Samples were obtained 8 h after starting infusion of physiological saline or LPS. LPS was mixed with tPA (3 mg/kg)

The 15-min infusion group received tPA/LPS mixture at a rate of 4 mL/h for 15 min, then switched to LPS-only infusion from 15 min to 4 h

The 4-h group received tPA/LPS mixture infused at a rate of 2.5 mL/h for 4 h

The 8-h group received tPA/LPS mixture infused at a rate of 2.5 mL/h for 4 h, then switched to tPA-only infusion at a rate of 0.5 mL/h for 4 h

^a^*P* < 0.05, showing a significant difference between the LPS group and the Control group

^b^*P* < 0.05, showing a significant difference between the LPS group and each of the LPS + tPA treatment group

### Therapeutic administration of tPA in LPS models

**Fig. 3:**
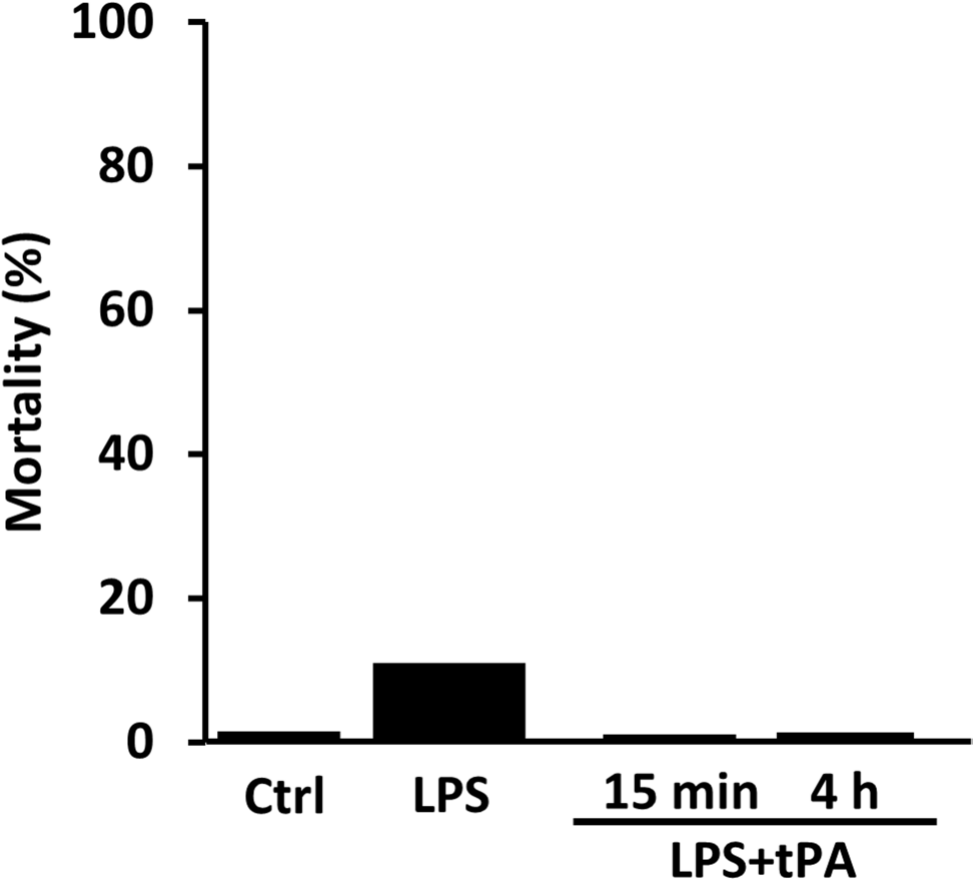
Changes in mortality at 8 h in the LPS-induced DIC model treated with therapeutic tPA. LPS was infused for a 4 h period (from 0 to 4 h). No significant differences were observed between the tPA-treated group and the LPS group. The 15-min infusion group received tPA at rate of 4 mL/h from 4 to 4 h 15 min. The 4-h infusion group received tPA at rate of 0.5 mL/h from 4 to 8 h.

The results of therapeutic tPA administration are shown in Table 2. The decrease in platelet counts was significantly suppressed in the LPS + tPA 3 mg/kg/15 min group (THER-tPA/15 min) and the LPS + tPA 3 mg/kg/4 h group (THER-tPA/4 h) compared with LPS group. Fibrinogen levels did not differ significantly in the THER-tPA/15 min or THER-tPA/4 h groups compared with the LPS group. PAI-1 activity was significantly lower in the THER-tPA/4 h group compared to LPS group. D-dimer levels were significantly higher in the THER-tPA/15 min and THER-tPA/4 h groups compared to LPS group. Similar to the trend observed with PAI-1, D-dimer levels progressively increased with longer tPA dosing time, despite the consistent tPA dose. TAT levels were significantly lower in the THER-tPA/15 min and THER-tPA/4 h groups compared to LPS group. Cr levels were significantly lower in the THER-tPA/15 min and THER-tPA/4 h groups compared to the LPS group. Levels of ALT did not differ significantly between THER-tPA/15 min and THER-tPA/4 h groups compared to the LPS group. The %GFD levels were significantly lower in the THER-tPA/15 min and THER-tPA/4 h groups compared to the LPS group. Levels of IL-6 tended to be lower in the THER-tPA/15 min and THER-tPA/4 h groups compared to the LPS group, but the difference was not significant. Urinary Hb levels were not significantly different in the THER-tPA/15 min or THER-tPA/4 h group compared to the LPS group. Mortality tended to be lower in the THER-tPA/15 min and THER-tPA/4 h groups compared to the LPS group, but the difference was not significant (Fig. 3).

**Table 2:** Changes in coagulation parameters, organ dysfunction and inflammatory cytokines in LPS treated with therapeutic tPA groups

Hemostatic variables	Control	LPS	LPS + tPA (3 mg/kg/15 min)	LPS + tPA (3 mg/kg/4 h)
Platelets, × 10^3^/µL	511 (409–561)	112 (40–240)^a^	224 (92–307)^b^	194 (137–379)^b^
Fibrinogen, mg/dL	204 (176–234)	71 (50–85)^a^	72 (50–108)	55 (50–126)
PAI-1, µg/L	9.1 (1.0–44.5)	859.9 (503.4–1769.1)^a^	630.3 (372.7–1190.4)	0.2 (0.1–6.8)^b^
D-dimer, mg/L	0.20 (0.20–0.20)	0.81 (0.57–1.27)^a^	3.55 (1.01–16.27)^b^	15.87 (0.93–24.44)^b^
TAT, µg/L	18 (6–50)	304 (262–402)^a^	236 (216–298)^b^	206 (104–266)^b^
Cr, mg/dL	0.22 (0.18–0.26)	0.44 (0.36–0.56)^a^	0.37 (0.32–0.40)^b^	0.33 (0.25–0.51)^b^
ALT, U/L	63 (25–113)	146 (75–223)^a^	131 (83–959)	75 (50–367)
%GFD, %	0 (0–5)	53 (15–80)^a^	10 (5–25)^b^	8 (0–20)^b^
IL-6, ng/L	82 (30–409)	61,220 (29,624–160,703)^a^	42,435 (29,643–52,462)	43,478 (11,865–70,280)
Urinary Hb, g/dL	0.0 (0.0–0.1)	0.0 (0.0–0.1)	0.0 (0.0–0.2)	0.0 (0.0–0.2)

Data are presented as median (min–max). Each group consisted of 7–8 animals

PAI-1, plasminogen activator inhibitor; TAT, thrombin–antithrombin complex; Cr, creatinine; ALT, alanine aminotransferase; IL-6, interleukin -6

Samples were obtained 8 h after starting infusion of physiological saline or LPS

The 15-min infusion group received tPA at rate of 4 mL/h from 4 to 4 h 15 min

The 4-h infusion group received tPA at rate of 0.5 mL/h from 4 to 8 h

^a^*P* < 0.05, showing a significant difference between the LPS group and the Control group

^b^*P* < 0.05, showing a significant difference between the LPS group and each of the LPS + tPA treatment group

## Discussion

Since DIC is primarily characterized by significant procoagulant activation, the fundamental therapeutic approach involves inhibiting procoagulant activation using anticoagulant drugs, alongside treatment of the underlying pathology [1, 2]. Conversely, the research and therapeutic advancements targeting fibrinolysis have been limited.

Several studies have investigated the effectiveness of fibrinolytic treatment in cases of clinical sepsis and models of DIC [16–18]. However, the number of reports is significantly smaller compared to those dealing with anticoagulation treatment for DIC, and no clear consensus has been reached. When tPA was administered to 62 children with meningococcal purpura fulminans, a condition known to be frequently associated with severe DIC, intracranial hemorrhage occurred in 8% of patients [16]. Simple fibrinolytic therapy for DIC is thus not considered the typical approach due to the risk of hemorrhage.

The degree of fibrinolytic activation in DIC varies significantly depending on the underlying pathology. In DIC caused by sepsis or severe infection, fibrinolysis is often markedly suppressed by inflammatory cytokines and endotoxins, leading to ischemic organ damage due to multiple microthrombi [3, 4]. Correction of suppressed fibrinolytic mechanisms may lead to recovery of the DIC pathophysiology. We, therefore, investigated the effects of tPA, which is highly thrombolytic in thrombotic disorders [19, 20], on the pathophysiology of suppressed fibrinolytic-type DIC (LPS-induced DIC model). Our previous studies have shown that prolonged prophylactic use of tPA (specifically, LPS at 0 -4 h, tPA 3 mg/kg at 0 -4 h, or tPA 6 mg/kg at 0 -8 h), such as tPA at 3 mg/kg/4 h or 6 mg/kg/8 h, clearly improved the DIC pathophysiology [13]. However, results from tPA at 3 mg/kg/4 h or 6 mg/kg/8 h were unclear regarding whether the lower doses were effective and whether prolonged administration was effective. Therefore, the present study added not only tPA at 3 mg/kg/4 h but also tPA at 3 mg/kg/15 min and 3 mg/kg/8 h in experiments on prophylactic tPA administration, to clarify whether efficacy at a constant dose varies with the duration of administration. In addition, for a closer approximation of clinical situations, we added an experiment with a therapeutic dosage of tPA following creation of the DIC model using LPS.

The LPS-induced DIC model is characterized by a significant increase in PAI-1 leading to fibrinolytic inhibition [21–24]. In this study, tPA exhibited consistent effects on PAI-1 activity in both experimental groups, whether prophylactic or therapeutic. That is, even with administration at the same tPA dosage, PAI-1 activity decreased in a time-dependent manner after tPA administration. Blood D-dimer, reflecting thrombolytic activity, also rose with prolonged tPA administration. The progressive decline in PAI-1 activity and the concurrent rise in D-dimer levels over time following tPA administration, despite consistent tPA dose, were considered to be interrelated phenomena. However, in the PROP-tPA/4 h group, a significant increase in PAI-1 activity was observed, and an increasing trend was also noted in the PROP-tPA/15 min group. Because PAI-1 activity increased from the end of tPA administration to the endpoint (8 h after the initiation of LPS administration), a rebound elevation of PAI-1 activity following the cessation of tPA infusion was considered possible. In addition, the simultaneous increase in D-dimer in the PROP-tPA/4 h group is difficult to reconcile with this finding based on the mechanism of fibrinolytic activation. As the precise causes of these observations remain unclear, further investigation is warranted.

Interestingly, prolonged administration of tPA, whether administered prophylactically or therapeutically, not only corrected the previously mentioned fibrinolytic suppression, but also reduced blood TAT levels, thereby reducing coagulation activation. The reason for this phenomenon is unknown, but we hypothesized that the decrease in TAT levels in the LPS model could be due to reduced thrombin production or to potentially being attributable to the time-dependent decrease in antithrombin universally observed in DIC[25]. In sepsis-induced DIC with suppressed fibrinolysis, crosstalk between coagulation activation and inflammation has been reported [26–29]. Inflammation induces coagulation activation. In addition, thrombin and active factor X, resulting from coagulation activation, induce the expression of proinflammatory cytokines via protease activated receptors [30, 31]. The coagulative and inflammatory processes are thus believed to mutually activate each other, emphasizing the importance of suppressing both reactions. In the present study, prophylactic administration of tPA showed a trend toward reducing blood IL-6 at 8 h. Ischemic heart disease and cerebral ischemia are associated with increased expressions of inflammatory cytokines such as IL-6 [32, 33]. In this study, tPA dissolved microthrombi in the blood vessels and prevented organ ischemia, potentially leading to decreased in IL-6 levels.

Further, an intriguing observation was the inhibition of decreases in platelet counts, particularly following prophylactic or therapeutic tPA administration. This was attributed to decreased thrombus formation leading to reduced platelet consumption.

Prophylactic or therapeutic tPA administration was associated with suppression of blood creatinine elevations. A significant decrease in %GFD was apparent, following extended tPA administration. This was attributed to the tPA dissolving microthrombi in renal glomeruli, resulting in concomitant improvements to renal damage.

Hemorrhagic symptoms are major side effects of tPA. Interestingly, no trend was seen toward increased urinary Hb or any hemorrhagic symptoms observed with tPA administration. This was attributed to lower doses of tPA, which were less likely to cause bleeding symptoms compared to those used in clinical practice, and to the correction of consumptive coagulopathy by tPA administration [14]. Nevertheless, the risk of bleeding significantly increases when tPA is administered for more than 46 h [34], highlighting the necessity for further studies to establish an upper limit on the administration time. On the other hand, in the present study, bleeding tendency was assessed only by measuring urinary Hb, which raises concerns about insufficient evaluation. Further detailed assessments, such as tail-cut analysis, will be necessary in future studies.

Among the parameters that exhibited improvement with tPA administration in this study, platelet count, fibrinogen, D-dimer, PAI-1 activity, TAT, IL-6, creatinine, and renal glomerular fibrin deposition demonstrated significant enhancements with prolonged administration. At least for ALT, no exacerbation was observed with prolonged administration. These findings strongly indicate that, in our experimental model, tPA improved DIC pathophysiology not by increasing the dosage, but by prolonging administration at the same dosage.

Similar results were obtained in the current experiment with both prophylactic and therapeutic tPA administration. When considering future clinical applications, prophylactic tPA administration is impractical in real-world clinical settings. Consequently, the similarity in results obtained with therapeutic tPA administration was deemed highly significant. However, as mentioned earlier, the significance of further extensions to the duration of tPA administration remains a subject for future investigation, and consideration of both risks and benefits is needed. In addition, tPA therapy for DIC should be reserved exclusively for suppressed-fibrinolytic-type DIC, particularly that is associated with sepsis. This approach should never be used for enhanced-fibrinolytic-type DIC cases. In short, the concept of DIC classification is essential when devising tPA treatment strategies [3, 4].

This study has several issues that should be considered in relation to its potential clinical application. First, for the therapeutic administration groups, the effect of tPA administration on improving mortality requires further evaluation. In the present study, mortality tended to decrease with therapeutic tPA administration, although no statistically significant difference was observed. This may have been due to the limited number of cases available for evaluating mortality. Second, the timing of tPA administration in the therapeutic administration group warrants investigation. In this study, tPA was administered immediately after completion of LPS administration. However, in clinical practice, it will be necessary to assess the efficacy when the interval between the end of LPS exposure and the start of tPA administration is varied. Third, plasmin activity was not assessed. To confirm the neutralization of PAI induced by continuous tPA infusion, it is necessary to evaluate plasmin activity, and this remains an important issue for future investigation.

In conclusion, tPA administration in the LPS-induced DIC model not only activates the fibrinolytic mechanism but also suppresses coagulation activation, leading to the amelioration of DIC pathophysiology. Fibrinolytic therapy with tPA, traditionally employed solely for treating thrombotic diseases, may emerge as a novel therapeutic approach for suppressed-fibrinolytic-type DIC, such as that induced by sepsis. Our study revealed that continuous low-dose tPA administration, even at the same dosage, for suppressed-fibrinolysis-type DIC, may rectify the state of fibrinolytic suppression and improve DIC pathophysiology.

## Data Availability

The data supporting the findings of this study are available from the corresponding author upon reasonable request.
